# The Differences between NAD-ME and NADP-ME Subtypes of C_4_ Photosynthesis: More than Decarboxylating Enzymes

**DOI:** 10.3389/fpls.2016.01525

**Published:** 2016-10-13

**Authors:** Xiaolan Rao, Richard A. Dixon

**Affiliations:** ^1^BioDiscovery Institute and Department of Biological Sciences, University of North Texas Denton, TX, USA; ^2^BioEnergy Science Center, US Department of Energy Oak Ridge, TN, USA

**Keywords:** C_4_ photosynthesis, C_4_ plants, NAD-ME subtype, NADP-ME subtype, comparative transcriptome analysis

## Abstract

As an adaptation to changing climatic conditions that caused high rates of photorespiration, C_4_ plants have evolved to display higher photosynthetic efficiency than C_3_ plants under elevated temperature, high light intensities, and drought. The C_4_ plants independently evolved more than 60 times in 19 families of angiosperms to establish similar but not uniform C_4_ mechanisms to concentrate CO_2_ around the carboxylating enzyme Rubisco (ribulose bisphosphate carboxylase oxygenase). C_4_ photosynthesis is divided into at least two basic biochemical subtypes based on the primary decarboxylating enzymes, NAD-dependent malic enzyme (NAD-ME) and NADP-dependent malic enzyme (NADP-ME). The multiple polygenetic origins of these subtypes raise questions about the association of C_4_ variation between biochemical subtypes and diverse lineages. This review addresses the differences in evolutionary scenario, leaf anatomy, and especially C_4_ metabolic flow, C_4_ transporters, and cell-specific function deduced from recently reported cell-specific transcriptomic, proteomic, and metabolic analyses of NAD-ME and NADP-ME subtypes. Current omic analysis has revealed the extent to which component abundances differ between the two biochemical subtypes, leading to a better understanding of C_4_ photosynthetic mechanisms in NAD-ME and NADP-ME subtypes.

## Introduction

In the warm-climate zones, C_4_ plants occupy nearly all grasslands and are a major component of the flora and biomass production through their improved photosynthetic, water and nutrient-use efficiencies (Sage, 2004; Gowik and Westhoff, 2011). C_4_ photosynthesis in C_4_ plants is not a single metabolic pathway. It has been established by a series of biochemical and morphological modifications to concentrate CO_2_ at the site of ribulose bisphosphate carboxylase oxygenase (Rubisco; Sage, 2004). In all C_4_ plants, CO_2_ is initially fixed by phosphoenolpyruvate (PEP) carboxylase. The resulting four-carbon acids are transported to an interior compartment where Rubisco is localized. Here, CO_2_ is released by a decarboxylating enzyme specific for the four carbon acid, and assimilated by Rubisco through the Calvin cycle. The decarboxylation reaction also produces a three-carbon acid, which diffuses back to the compartment where PEP carboxylase is located (Hatch, 1987; Sage, 2004; Sommer et al., 2012). Almost all C_4_ plants require the coordination of mesophyll (M) and bundle sheath (BS) cells (called Kranz anatomy) to separate primary and secondary carbon fixation reactions, while a few exceptions use internal subcellular compartmentalization within a single cell (Hatch, 1987; Offermann et al., 2015).

Historically, C_4_ photosynthesis in traditional text books has been classified into three subtypes based on the predominant decarboxylating enzymes of the four carbon acid, NAD-dependent malic enzyme (NAD-ME), NADP-dependent malic enzyme (NADP-ME), and PEP carboxykinase (PEPCK) (Hatch, 1987). However, multiple pieces of evidence challenge the establishment of the PEPCK subtype; no pure PEPCK-type C_4_ species has been discovered (Sage, 2004), and the robust model analysis of “pure PEPCK type” indicates the imbalance of energy requirements in BS and M cells (Wang et al., 2014). Therefore, currently NAD-ME and NADP-ME subtypes are suggested as distinct C_4_ biochemical pathways, both with or without the additional service of the PEPCK pathway. Many productive cereal, forage, and biofuel crops belong to either the NADP-ME C_4_ subtype, for example, maize (*Zea mays*), sugarcane (*Saccharum* spp.), and sorghum (*Sorghum bicolor*), or to the NAD-ME subtype, for example, switchgrass (*Panicum virgatum* L.), pearl millet [*Pennisetum glaucum* (L.) R. Br], and amaranth (Amaranthaceae) (Edwards and Walker, 1983).

During the past decade, high throughput tools have made it possible to quantify the transcriptome, proteome, and metabolome at the cell- or tissue-levels (Metzker, 2010). Such applications have expanded the borders and enhanced our knowledge of C_4_ photosynthesis, which was first reported in the 1950s. In this review, we focus on the differences associated with C_4_ photosynthesis in NAD-ME and NADP-ME subtypes in terms of genetic, physiological, cytological, biochemical, and molecular traits.

## Evolutionary Scenarios of NAD-ME and NADP-ME Subtypes

The evolution of C_4_ photosynthesis has been achieved over 60 times through individually adaptive steps in 19 families of angiosperms (Sage, 2004), and was hypothetically triggered by the decrease of atmospheric CO_2_ concentration and plant hydraulics (Christin et al., 2008; Osborne and Sack, 2012). The NAD-ME and NADP-ME subtypes represent almost equal numbers of genera in the eudicots, and the NADP-ME subtype dominates in monocot families (Sage et al., 2011).

The distinct subtypes and lineages of C_4_ plants were hypothesized to have evolved in adaptation to selective pressure such as shortage of nitrogen and water (Liu and Osborne, 2015; Brautigam and Gowik, 2016). Global geographic surveys of C_4_ grasses have shown that the NAD-ME subtype occurs more in drier areas and the percentage of NADP-ME subtypes increases with annual precipitation (Vogel et al., 1978; Hattersley, 1992; Taub, 2000). Correspondingly, the largely NAD-ME grass lineage Chloridoideae exhibits a significantly greater enhancement of water use efficiency than NADP-ME grasses under drought condition, due to its leaf structure and faster leaf curling rates (Ghannoum et al., 2002; Liu and Osborne, 2015). High correlation was observed between photosynthetic nitrogen use efficiency and the NADP-ME subtype. Plants in the NADP-ME subtype (except those in the Aristidoideae tribe) tend to have higher photosynthetic nitrogen use efficiency compared with other C_4_ grasses under adequate or deficient nitrogen supply (Taub and Lerdau, 2000; Ghannoum, 2005; Pinto et al., 2014, 2015). A reduced content of nitrogen and faster Rubisco activity in leaves contribute to better nitrogen-use efficiency in NADP-ME grasses (Ghannoum, 2005). However, the association of C_4_ subtypes with particular physiological traits, and whether the optimization of nitrogen or water usage drives the evolution of at least some C_4_ lineages, remain to be determined.

**FIGURE 1:**
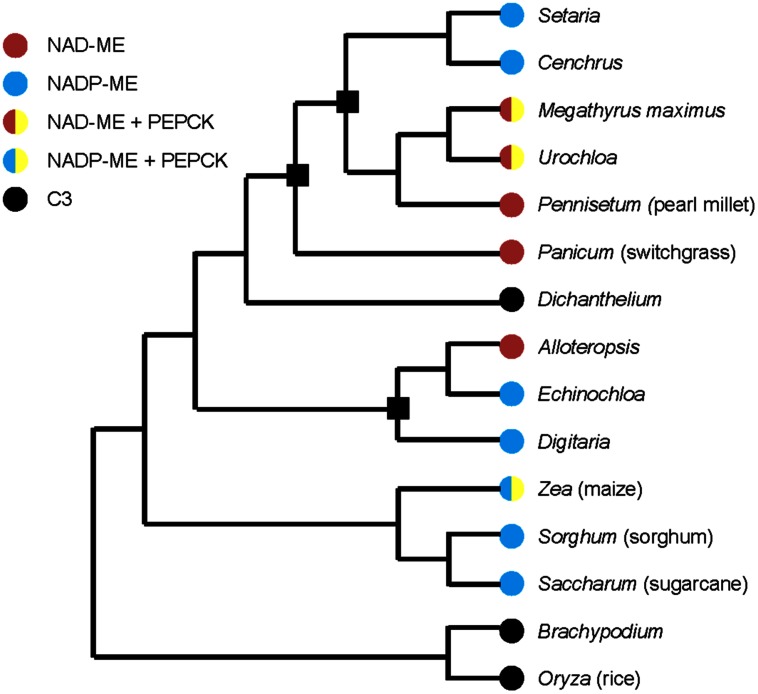
**Simplified phylogenetic tree of grasses with selected C_4_ and C_3_ families, drawn based on Grass Phylogeny Working Group II (2012) and Washburn et al. (2015).** Black square means the possible evolutionary conversions between NAD-ME and NADP-ME subtypes. Representative species of families are marked in parentheses.

Multiple origins of C_4_ photosynthesis have been suggested as C_3_ to C_4_ transitions and evolutionary conversions between two C_4_ subtypes (Grass Phylogeny Working Group II, 2012; Washburn et al., 2015). **Figure 1** shows a simplified example of a phylogenetic tree with selected families of grasses and three origins (black squares in **Figure 1**) are considered as the evolutionary conversions between NAD-ME and NADP-ME subtypes (Grass Phylogeny Working Group II, 2012; Washburn et al., 2015). Three models have been proposed to describe the evolutionary divergence of C_4_ subtypes. One places the NAD-ME subtype as the ancestral C_4_ subtype, with the NADP-ME subtype evolving from it (Gutierrez et al., 1974; Washburn et al., 2015). The second model proposes that the NAD-ME and NADP-ME subtypes were shared at some level in a common C_4_ ancestor, then individually predominated in distinct lineages (Washburn et al., 2015). In the third model, NAD-ME and NADP-ME subtypes evolved independently from a C_3_–C_4_ intermediate as their most recent common ancestor (Washburn et al., 2015).

The evolutionary transition to C_4_ photosynthesis remains undetermined. Phylogenomics analysis has indicated the difference of recruitment of major decarboxylating enzymes into C_4_ photosynthesis in NAD-ME and NADP-ME subtypes (Maier et al., 2011; Christin et al., 2013). The non-photosynthetic NAD-ME in C_3_ plants is composed of α and β subunits, and functions as a homodimer and heterodimer in the respiration of malate in mitochondria of all cells (Tronconi et al., 2008). In leaves of the dicot C_4_ plant *Cleome gynandra*, transcripts corresponding to two genes encoding α and β subunits are abundant in BS cells (Brautigam et al., 2011; Aubry et al., 2014) and the formation of heterodimeric photosynthetic NAD-ME was found in leaves of *Amaranthus hypochondriacus* (Long et al., 1994), whereas in the monocot C_4_ plant switchgrass, only one gene encoding the NAD-ME β subunit is highly expressed in BS cells of leaves (Rao et al., 2016), and an octamer of only one type of subunit exists in *Eleusine coracana* and *Panicum dichotomiflorum* leaves (Murata et al., 1989). Phylogenetic analysis indicates that NAD-MEs in C_4_ plants evolved from the existing mitochondrial NAD-ME and may be acquired through changes in regulatory and kinetic properties, rather than gene duplication (Maier et al., 2011). In contrast, C_4_ NADP-ME, which is thought to derive from a C_3_ chloroplast-localized ancestor and rooted from an ancient cytosolic isoform, has specific function in C_4_ photosynthesis in BS cells of NADP-ME subtype plants (Maier et al., 2011).

The emergence of C_4_ NAD-ME and NADP-ME would include the steps of enriched expression in BS cells and optimization of enzymatic properties (Maier et al., 2011). However, the preferential expression of NAD-ME and NADP-ME may be not exclusively correlated with its corresponding subtype. Significant transcript levels of genes associated with NAD-ME subtype C_4_ photosynthesis and high NAD-ME activity have been observed in the C_3_–C_4_ intermediate species *Flaveria ramosissima*, which is close to the NADP-ME C_4_ *Flaveria* lineage (Gowik et al., 2011). Additionally, the NADP-ME ortholog was found to be preferentially accumulated in BS cells of NAD-ME subtype switchgrass, in which low NADP-ME activity was detected (Rao et al., 2016). These unpredicted transcript profiles may reflect the common C_4_ ancestor, within which NAD-ME and NADP-ME subtype C_4_ pathway are present together at some level (Gowik et al., 2011; Washburn et al., 2015).

## Kranz Anatomy in Leaves of NAD-ME and NADP-ME Subtypes

In most C_4_ species, an altered arrangement of cells within the leaf known as Kranz anatomy facilitates the cellular compartmentation of carboxylation and decarboxylation (Nelson and Langdale, 1992; Heckmann, 2016). A typical Kranz anatomy includes an outer layer of chloroplast-containing M cells for initial carboxylation, and an inner layer of large, distinctive BS cells that surround the vascular bundle for carbon reduction (Sage, 2004).

Kranz form varies as a consequence of the distinct evolutionary origins of C_4_ plants (Fouracre et al., 2014). In the NADP-ME subtype, the layer of cells between the BS cells and the vascular bundle is absent, and suberin is deposited in the BS cell wall. BS chloroplasts with reduced grana are arranged centrifugally in monocotyledons and centripetally in dicotyledons (Gutierrez et al., 1974; Hattersley and Watson, 1976; Prendergast et al., 1987; Lundgren et al., 2014). Comparatively, the vasculature of the NAD-ME subtype is usually surrounded by a double sheath, consisting of the outer BS and the inner non-photosynthetic mestome sheath (Prendergast et al., 1987; Lundgren et al., 2014). Suberin ubiquitously deposits in the mestome sheath rather than in BS cells, and BS chloroplasts with developed grana are arranged centripetally (Hattersley and Watson, 1976; Nelson and Langdale, 1989; Fouracre et al., 2014; Mertz and Brutnell, 2014). Loss of one layer of mestome sheath cells in the NADP-ME type suggests differences in the origination of cell divisions. The single BS in C_4_ NADP-ME type grasses is derived from the procambium and M cells develop from the ground meristem. In the double-sheath species of the NAD-ME type, both the BS and M cells are derived from the ground meristem and the mestome sheath is derived from the procambium (Nelson and Langdale, 1989; Soros and Dengler, 2001).

## Metabolite Flow of C_4_ Photosynthesis in NAD-ME and NADP-ME Subtypes

**FIGURE 2:**
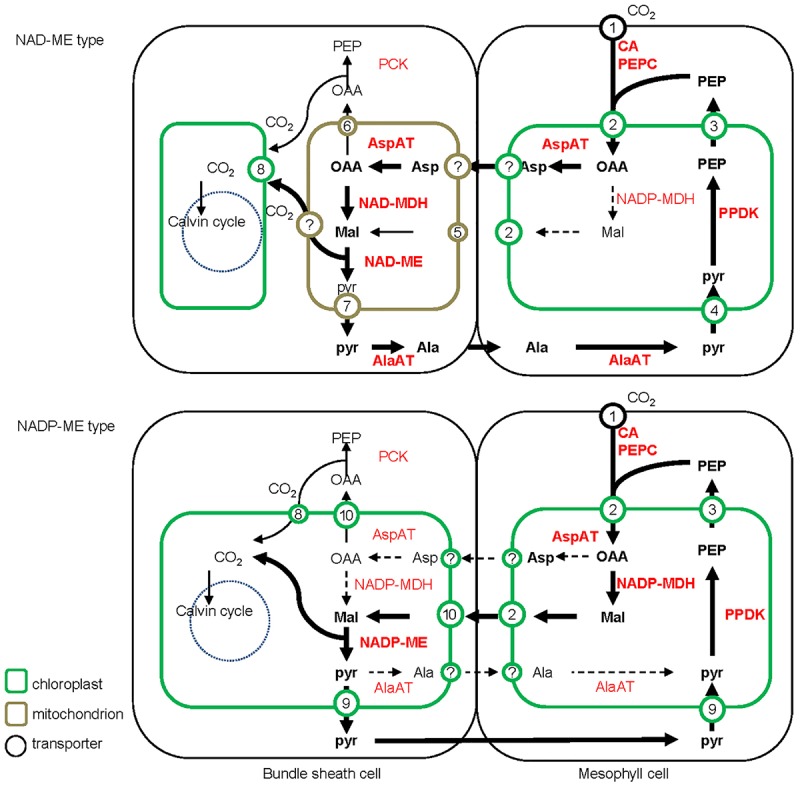
**Detailed schematic of the C_4_ photosynthesis pathway of NAD-ME and NADP-ME subtypes.** The major C4 biochemical pathway, the additional PEPCK pathway and the possible alternative pathway are indicated with bold, narrow, and dashed lines, respectively. The abundances of 4-carbon acids (metabolite level) and transporters (transcript level) are indicated with font style (with bold representing more abundant) and font/circle size (with larger representing more abundant), respectively. Ala, alanine; Asp, aspartate; Mal, malate; Pyr, pyruvate; OAA, oxaloacetate; PEP, phosphoenolpyruvate; CA, carbonic anhydrase; PEPC, phosphoenolpyruvate carboxylase; PPDK, pyruvate/orthophosphate dikinase; AspAT, aspartate aminotransferase; AlaAT, alanine aminotransferase; NADP-MDH, NADP-dependent malate dehydrogenase; NADP-ME, NADP-dependent malic enzyme; NAD-MDH, NAD-dependent malate dehydrogenase; NAD-ME, NAD-dependent malic enzyme; PCK, phosphoenolpyruvate carboxykinase. 1, Plasma membrane intrinsic protein (PIP); 2, dicarboxylate transporter 1 (DiT1, OMT1); 3, phosphate/phosphoenolpyruvate translocator (PPT); 4, sodium bile acid symporter 2 (BASS2) and sodium:hydrogen antiporter (NHD); 5, malate phosphate antiport 1 (DIC1) and phosphate proton symport (PIC); 6, mitochondrial carrier (DTC); 7, mitochondrial pyruvate carrier (MPC); 8, plasma membrane intrinsic protein (PIP) of chloroplast; 9, proton:pyruvate cotransporter (MEP); 10, dicarboxylate transport 2 (DiT2, DCT2).

All C_4_ plants share a common enzymatic step, the initial carboxylation reaction catalyzed by PEPC to yield oxaloacetic acid (OAA) in M cells (Sage, 2004). Subsequent steps to concentrate CO_2_, the transported metabolites, and the subcellular localization of the decarboxylation reaction, differ between the different biochemical subtypes (**Figure 2**).

The traditional biochemical view of the NADP-ME subtype places malate, derived from OAA, as the dominant transported metabolite to diffuse to the BS cells. Pyruvate is formed during the decarboxylation reaction, and returns to the M cells to be phosphorylated back to PEP. The synthesis of malate occurs in the M chloroplasts and the decarboxylation by NADP-ME in the BS chloroplasts. In contrast, NAD-ME plants use aspartate as the major transport metabolite, which is formed by transamination of OAA. After transfer to the BS cells, aspartate is converted to malate by a reductive deamination reaction. Pyruvate is also formed during the NAD-ME decarboxylation reaction, but is partially transported back to the M cells in the form of alanine to maintain the ammonia balance between the two cell types. Alanine in the M is converted through several steps into PEP, which provides the precursor for a new round of carboxylation and decarboxylation.

In the NAD-ME subtype, aspartate is synthesized in the M cytosol, while malate formation and decarboxylation by NAD-ME occur in the BS mitochondria (Hatch, 1971; Edwards and Walker, 1983; Hatch, 1987; Weber and von Caemmerer, 2010; **Figure 2**). In addition, the activity of PEPCK enzyme was detected at different levels in multiple lineages of NAD-ME and NADP-ME subtypes, which can decarboxylate OAA to PEP for CO_2_ release in the cytosol of BS cells (Pick et al., 2011; Sage et al., 2011; **Figure 2**). The supplementary utilization of the PEPCK pathway in some C_4_ plants is considered to enhance plant adaption to various environmental conditions (Wang et al., 2014; Furbank, 2016).

However, flexibility in the NADP-ME type C_4_ carbon fixation mechanism has been observed. Early C^14^ labeling experiments in maize (NADP-ME type C_4_ monocot) showed that label was incorporated into both malate and aspartate, with the latter occupying a minor but significant proportion (approximately 25%) of the active C_4_ acid pool (Hatch, 1971). Subsequent experiments showed that, in the presence of 2-oxoglutarate, aspartate can be decarboxylated by isolated BS cells of maize at lower rates (Chapman and Hatch, 1981). A similar study in *Flaveria bidentis* (NADP-ME type C_4_ dicotyledon) revealed that aspartate and malate contributed equally to transfer CO_2_ to the BS cells (Meister et al., 1996). High levels of transcripts encoding the major isoforms of aspartate aminotransferase (AspAT) and alanine aminotransferase (AlaAT) were detected in maize leaves, and enzymatic assays further confirmed the sufficiency of aminotransferase activity to carry out the carboxylation and decarboxylation reaction (Pick et al., 2011). Interestingly, cell-specific transcriptome analysis has revealed that AspAT and AlaAT are preferentially expressed at high levels in M and BS cells, respectively, in both NADP-ME type maize and *Setaria viridis* (Chang et al., 2012; John et al., 2014). The preferential accumulation of AspAT and AlaAT proteins was confirmed by proteomics analysis in isolated M (Majeran et al., 2005) and BS (Manandhar-Shrestha et al., 2013) cells of maize.

Several hypotheses have been put forward to explain how aspartate contributes to carbon fixation in NADP-ME plants. Majeran et al. (2005) suggested that the abundance of AspAT in M chloroplasts serves as a metabolic link between amino acid synthesis and nitrogen assimilation to generate aspartate as the final step of incorporation of ammonia into amino acid. This view is consistent with the observation of accelerated turnover of aspartate in response to nitrogen deficiency in maize leaves (Khamis et al., 1992). The reduction of cellular aspartate may decrease the rate of protein synthesis, and transcriptome analysis has indeed revealed reduced expression of protein synthesis-related genes in some NADP-ME plants (Brautigam et al., 2011; Gowik et al., 2011). This likely causes the reduction of protein content and therefore higher nitrogen-use efficiency in NADP-ME plants, compared with that of their NAD-ME counterparts (Brautigam and Gowik, 2016).

The fate of aspartate after translocation from M to BS cells is unclear. One proposal is that it can serve as a C_4_ regulator by influencing the transport of malate or pyruvate across the BS chloroplast, rather than serving a metabolic role (Chapman and Hatch, 1979). Another proposal is that AspAT in BS cells converts aspartate into OAA. OAA can be directly decarboxylated in the cytosol by PEP-CK (route I), or re-reduced to malate and then decarboxylated by NADP-ME in the chloroplast (route II) (Furbank, 2011; Gowik and Westhoff, 2011; Pick et al., 2011). However, for route I, no or limited PEP-CK activity has been reported in some NADP-ME subtype plants such as *S. bicolor* and *F. bidentis*; for route II, the mixed model including four transfer acids (aspartate, malate, alanine, and pyruvate) of the NADP-ME subtype requires comparable amounts of AspAT and AlaAT in BS cells to those in M cells, which is not consistent with the finding of the unequal accumulation of aminotransferase in M and BS chloroplasts in maize (Majeran et al., 2005; Manandhar-Shrestha et al., 2013). Rigid definitions of decarboxylation pathways may be misleading, and variants of the C_4_ NADP-ME subtype may be considered (Wang et al., 2014). Aspartate may be transaminated and decarboxylated by PEP-CK in NADP-ME variants that present sufficient PEP-CK activity, such as maize, or be transformed into the donor of NADP-ME in NADP-ME variants such as *F. bidentis*, which has substantial PSII activity in BS to maintain redox balance during the reduction of aspartate (Meister et al., 1996).

## Plastid Transporters Involved in C_4_ Photosynthesis in NAD-ME and NADP-ME Subtypes

The dispersed sub-localization of carboxylating, decarboxylating, and transaminase enzymes in M and BS cells of C_4_ plants requires the collaboration of multiple translocators to transfer reaction substrates and products across membranes. NAD-ME and NADP-ME subtype plants utilize different plastid transports to maintain this metabolite flux (**Figure 2**).

In all C_4_ versions, pyruvate is predominantly found in M cells, where it is converted to PEP as the precursor for fixing CO_2_. Pyruvate in M cells is compartmented in chloroplasts making its cytosolic concentration low (Ohnishi et al., 1990). Two different mechanisms for transport of pyruvate into M chloroplasts have been identified in a range of C_4_ species: proton-dependent and sodium-dependent (Aoki et al., 1992; Furumoto et al., 2011), with the assumption that NAD-ME and NADP-ME types might use sodium:pyruvate and proton:pyruvate cotransporters, respectively (Ohnishi et al., 1990; Weber and von Caemmerer, 2010). Recent comparative transcriptome analyses between NAD-ME and NADP-ME type C_4_ plants have supported this hypothesis; transcripts encoding sodium:pyruvate cotransporter were preferentially expressed in M cells of the NAD-ME-type plants switchgrass and *C. gynandra*, whereas transcripts encoding proton:pyruvate cotransporter were enriched in M cells of the NADP-ME-type plants *S. viridis* and maize (Chang et al., 2012; Aubry et al., 2014; John et al., 2014; Rao et al., 2016).

The decarboxylation and assimilation of CO_2_ both happen in BS chloroplasts of NADP-ME type plants, and metabolite transporters are required to transfer malate and pyruvate across the chloroplast envelope membrane. The major decarboxylating enzyme NAD-ME, NAD-MDH, and AspAT are restricted to mitochondria in NAD-ME subtypes. Compared with NADP-ME subtypes, additional mitochondrial transporters are required in NAD-ME subtypes, including those for imported OAA and glutamate, exported aspartate and 2-oxoglutarate for the AspAT processes and imported malate and exported pyruvate for the NAD-MDH and NAD-ME processes. These postulated carriers involved in the C_4_ biochemical pathways are indicated on **Figure 2**.

A high rate of CO_2_ diffusion across the plasma membrane of M cells is expected in all C_4_ versions. Compared with NADP-ME subtype plants, an additional transport process is required to facilitate the CO_2_ permeability of BS chloroplasts in NAD-ME subtype plants since CO_2_ is released outside of the chloroplast. The membrane channel aquaporins, PIPs (plasma membrane intrinsic protein), have been demonstrated to mediate M CO_2_ conductance in leaves of some C_3_ plants such as tobacco (NtAQP1; Uehlein et al., 2008), *Arabidopsis* (AtPIP1;2; Uehlein et al., 2012), and barley (PIP2 family; Mori et al., 2014). The role of PIPs in CO_2_ diffusion is still unclear in C_4_ plants. The diurnal expression of ZmPIPs in the M of maize leaves might suggest their possible roles as CO_2_ facilitators (Hachez et al., 2008), and CgPIP1B was suggested to be the candidate CO_2_ transporter across the M cell plasmalemma in *Cleome* (Brautigam et al., 2011). PIPs, especially the PIP2 subfamily, also show high water transport activity (Katsuhara and Hanba, 2008) and the activity of PIPs is dynamically controlled in BS cells in *Arabidopsis* as a response to hydraulic stress (Shatil-Cohen et al., 2011). The dual roles of PIPs in CO_2_ and water transport might be responsible for CO_2_ assimilation and water movement in C_4_ plants, also contributing to resistance of C_4_ plants to drought stress. Furthermore, it is worth considering whether the additional service of PIPs in BS cells of the NAD-ME subtype increases drought tolerance, at least in some lineages.

## M and BS Cell-Specific Functions in NAD-ME and NADP-ME Subtypes

The spatial compartmentation of many metabolic pathways has been observed in M and BS cells of C_4_ plants (Majeran et al., 2005), and has generally been considered to be associated with the spatial separation of carboxylation and decarboxylation in the two cell types. There are both overlapping and differential cell-specific features of metabolic pathways in M and BS cells in NAD-ME and NADP-ME plants (Zhao et al., 2013; Koteyeva et al., 2014).

The light-dependent reactions of photosynthesis are not equally distributed in M and BS cells of NADP-ME plants. There is a depletion of PSII activity and reduction of the associated development of grana generally present at various degrees in BS chloroplasts of NADP-ME type species such as maize, sorghum, and sugarcane (Chapman and Hatch, 1981; Meierhoff and Westhoff, 1993). In contrast, enhancement of PSII activity and grana development in BS chloroplasts is observed in NAD-ME plants (Edwards and Walker, 1983). This is because, in the NADP-ME subtype, the primary shuttle of malate from M cells to BS chloroplasts provides NADPH, the balance of which would be influenced by a high level of PSII activity, whereas the transferred C_4_ acid aspartate in NAD-ME subtype does not deliver NADPH as reductive power (Edwards and Walker, 1983; Koteyeva et al., 2014).

Biochemical studies have further revealed differences in metabolic control of the Calvin cycle in BS cells of NAD-ME and NADP-ME subtypes; the addition of ribose-5-phosphate significantly increased light-dependent CO_2_ fixation, and light is required in C_4_ acid decarboxylation and assimilation into the Calvin cycle in maize (NADP-ME type), but ribose-5-phosphate only partially or little affected light-dependent CO_2_ fixation in *Atriplex spongiosa* and *Panicum miliaceum* (NAD-ME type; Hatch and Kagawa, 1976). This suggests that there would be insufficient supply of ribulose 1,5-diphosphate in the Calvin cycle and the ratio of C_4_ assimilation into the cycle might be controlled in NADP-ME subtype, whereas the Calvin cycle functions independently in NAD-ME subtype plants (Hatch and Kagawa, 1976).

Recently, comparative transcriptome analysis has indicated differential enrichment of transcripts involved in RNA regulation and protein biogenesis/homeostasis in M and BS cells of two NAD-ME-type plants (switchgrass and *Cleome*) and two NADP-ME-type plants (maize and *S. viridis*) (Chang et al., 2012; Aubry et al., 2014; John et al., 2014; Rao et al., 2016). Transcripts involved in protein synthesis, folding, and assembly are more abundant in M cells in the two NADP-ME-type plants, but are preferentially or equally expressed in BS cells of the two NAD-ME-type plants. In contrast, transcripts involved in RNA regulation are enriched in BS cells of the two NADP-ME-type plants, but are more abundant in M cells of the NAD-ME-type plant switchgrass. The differentiation for transcriptional and post-transcriptional regulatory mechanisms in M and BS cells of NADP-ME and NAD-ME types might be associated with the unequal distribution of metabolites within the M and BS cells of these two subtypes (Rao et al., 2016).

## Conclusion

A brief overview of the differences in features of NAD-ME and NADP-ME plants is shown in **Table 1**. C_4_ photosynthesis represents one of the most successfully evolutionary events in response to environmental change on the earth and can be divided into two broad biochemical groups, NAD-ME and NADP-ME. A clear statement of dichotomy in morphology and biochemistry can be made between the two C_4_ subtypes with some exceptions (Sage, 2004; Sage et al., 2011; Lundgren et al., 2014). The nature and commonality of C_4_ transporters and cell-type specific functional differentiation still remain to be determined beyond a few well-studied species, to explore whether these are common in most C_4_ plants or only within some C_4_ lineages. The diversification of physiological, biochemical, and molecular functions of the NAD-ME type and NADP-ME type might be a result of their distinct evolutionary pathways, and be associated with the accommodation of various environmental conditions.

**Table 1:** Summary of the different traits associated with NAD-ME and NADP-ME subtypes.

Traits	Description	NAD-ME	NADP-ME	Reference
Evolutionary scenario	Recruitment of NAD-ME or NADP-ME from C_3_ ancestor	NAD-ME comes from existed mitochondrial NAD-ME; dual performance in C_4_ photosynthesis and all cells	NADP-ME arises from gene duplication from C_3_ ancestor; specific function in C_4_ photosynthesis	Maier et al., 2011
Physiology		Higher water use efficiency (?)	Higher photosynthetic nitrogen use efficiency	Ghannoum, 2005; Pinto et al., 2014, 2015
Kranz anatomy	Chloroplast position in BS cells	Centrifugal	Centripetal in monocot centrifugal in dicot	Lundgren et al., 2014
	Grana in BS chloroplasts	Developed	Reduced	
	Inner layer of bundle sheath in BS cells	Double sheath (mestome sheath and vascular bundle)	Single sheath (vascular bundle)	
	The origination of cell division	BS and M derived from the ground meristem; the mestome sheath derived from the procambium	BS derived from the procambium; M cells derived from ground meristem	Soros and Dengler, 2001
C_4_ biochemical cycle	Enzymes and site of decarboxylation	NAD-malic enzyme in mitochondrion	NADP-malic enzyme in chloroplast	Furbank, 2011; Gowik and Westhoff, 2011; Pick et al., 2011
	Decarboxylated acid	Aspartate/alanine	Malate/pyruvate Aspartate/alanine (?)	
C_4_ plastid transporters	Pyruvate transport in chloroplasts of M cells	Sodium:pyruvate cotransporters	Proton:pyruvate cotransporters in monocots	Weber and von Caemmerer, 2010; Brautigam et al., 2011; Furumoto et al., 2011
	Transporters in mitochondrion of BS cells	Required	N/A	
	CO_2_ transport in chloroplasts of BS cells	Required	N/A	
M and BS cell-specific function	PSII activity in BS cells	Enhanced	Reduced	Meierhoff and Westhoff, 1993
	C_4_ acid decarboxylation and assimilation	Light-dependent; sufficient supply of ribulose-1,5-diphosphate in Calvin cycle	Partially light-dependent; insufficient supply of ribulose-1,5-diphosphate in Calvin cycle	Hatch and Kagawa, 1976
	Cell type-gene enrichment	RNA regulation enhanced or equally distributed in M cells; protein biogenesis enhanced or equally distributed in BS cells	RNA regulation enhanced in BS cells; protein biogenesis enhanced in M cells	Chang et al., 2012; Aubry et al., 2014, John et al., 2014; Rao et al., 2016

*The statements remaining in argument are indicated with question marks. BS, bundles sheath; M, mesophyll; N/A, not applied.*

## Author Contributions

XR collected data from literature and wrote the manuscript. RD revised the article.

## Conflict of Interest Statement

The authors declare that the research was conducted in the absence of any commercial or financial relationships that could be construed as a potential conflict of interest.
