# Corrigendum: The Differences between NAD-ME and NADP-ME Subtypes of C_4_ Photosynthesis: More than Decarboxylating Enzymes

**DOI:** 10.3389/fpls.2019.00247

**Published:** 2019-03-08

**Authors:** Xiaolan Rao, Richard A. Dixon

**Affiliations:** ^1^BioDiscovery Institute and Department of Biological Sciences, University of North Texas, Denton, TX, United States; ^2^BioEnergy Science Center, US Department of Energy, Oak Ridge, TN, United States

**Keywords:** C_4_ photosynthesis, C_4_ plants, NAD-ME subtype, NADP-ME subtype, comparative transcriptome analysis

In the original article, there was a mistake in Figure 2, as published. The names of the cell types, “mesophyll cell” and “bundle sheath” were erroneously interchanged. The corrected Figure 2 appears below.

**Figure 2:**
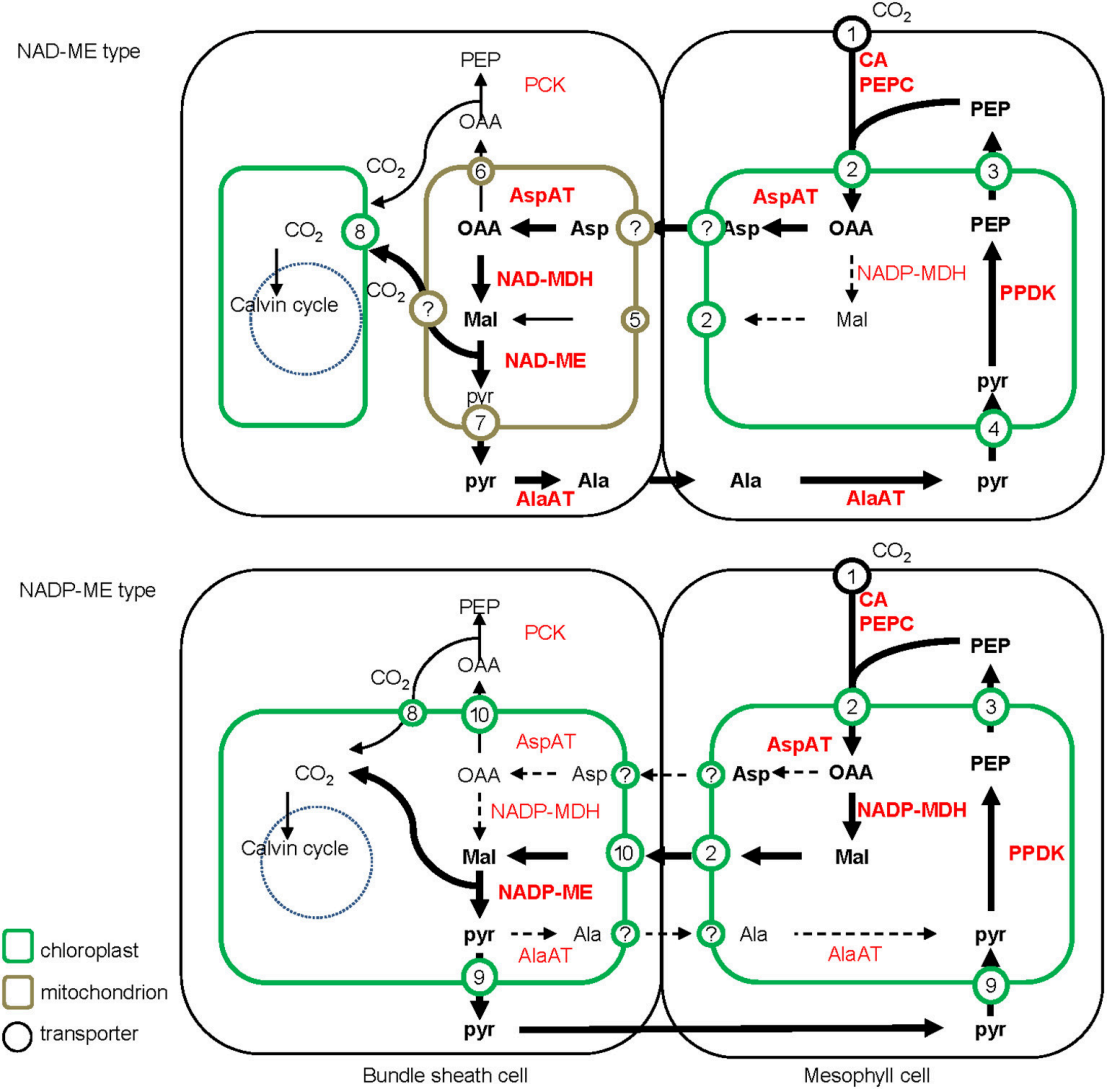
Detailed schematic of the C_4_ photosynthesis pathway of NAD-ME and NADP-ME subtypes. The major C4 biochemical pathway, the additional PEPCK pathway and the possible alternative pathway are indicated with bold, narrow, and dashed lines, respectively. The abundances of 4-carbon acids (metabolite level) and transporters (transcript level) are indicated with font style (with bold representing more abundant) and font/circle size (with larger representing more abundant), respectively. Ala, alanine; Asp, aspartate; Mal, malate; Pyr, pyruvate; OAA, oxaloacetate; PEP, phosphoenolpyruvate; CA, carbonic anhydrase; PEPC, phosphoenolpyruvate carboxylase; PPDK, pyruvate/orthophosphate dikinase; AspAT, aspartate aminotransferase; AlaAT, alanine aminotransferase; NADP-MDH, NADP-dependent malate dehydrogenase; NADP-ME, NADP-dependent malic enzyme; NAD-MDH, NAD-dependent malate dehydrogenase; NAD-ME, NAD-dependent malic enzyme; PCK, phosphoenolpyruvate carboxykinase. 1, Plasma membrane intrinsic protein (PIP); 2, dicarboxylate transporter 1 (DiT1, OMT1); 3, phosphate/phosphoenolpyruvate translocator (PPT); 4, sodium bile acid symporter 2 (BASS2) and sodium:hydrogen antiporter (NHD); 5, malate phosphate antiport 1 (DIC1) and phosphate proton symport (PIC); 6, mitochondrial carrier (DTC); 7, mitochondrial pyruvate carrier (MPC); 8, plasma membrane intrinsic protein (PIP) of chloroplast; 9, proton:pyruvate cotransporter (MEP); 10, dicarboxylate transport 2 (DiT2, DCT2).

The authors apologize for this error and state that this does not change the scientific conclusions of the article in any way. The original article has been updated.

